# Comprehensive Characterization of the Bioactive Profile in *Spirulina platensis* Vinegar

**DOI:** 10.3390/foods15122097

**Published:** 2026-06-11

**Authors:** Elif Yildiz, Ozan Gurbuz, Tugce Boga Demirel, Kubra Topaloglu Gunan, Metin Guldas

**Affiliations:** 1 Food Engineering Department, Faculty of Agriculture, Bursa Uludag University, Bursa 16059, Türkiye; ozang@uludag.edu.tr; 2 Gastronomy and Culinary Arts Department, Fine Arts Faculty, Maltepe University, Istanbul 34857, Türkiye; tugceboga@maltepe.edu.tr (T.B.D.); kubratopaloglu@maltepe.edu.tr (K.T.G.); 3 Nutrition and Dietetics Department, Health Sciences Faculty, Bursa Uludag University, Bursa 16059, Türkiye; mguldas@uludag.edu.tr

**Keywords:** *Spirulina platensis*, vinegar, fermentation, organic acid, phenolic compound, bioactivity, bioaccessibility, PCA

## Abstract

This study evaluated the bioactive properties of *Spirulina platensis*-based vinegar (SV) compared to apple vinegar (AV), focusing on organic acids, phenolic compounds, antioxidant capacity, and in vitro bioaccessibility. SV showed a significantly enhanced bioactive profile, with higher levels of key compounds such as gallic acid (86.99 ± 0.14 mg/L), succinic acid (15,859.43 ± 147.24 mg/L), and shikimic acid (147.13 ± 1.37 mg/L), indicating active fermentation-driven biotransformation. In addition, phytic acid present in *Spirulina powder* (494.43 ± 5.57 mg/L) was completely eliminated after fermentation. Importantly, SV exhibited significantly higher bioaccessible antioxidant capacity (25.68 ± 0.06 µmol TE/mL) than AV (8.68 ± 0.04 μmol TE/mL) based on the ABTS assay. Principal component analysis confirmed that organic acids were the main drivers of bioactive potential, while phenolics contributed to compositional differentiation. Overall, these results indicate that Spirulina platensis is a promising raw material for producing functional vinegar with enhanced bioactivity and bioaccessibility.

## 1. Introduction

Microalgae are rich in bioactive compounds that are promising for environmental sustainability. By efficiently utilizing sunlight, they provide a valuable source of numerous biological compounds such as proteins, carbohydrates, lipids, pigments, and carotenoids [1]. Aztec and Mayan civilizations used *Spirulina* (*Spirulina platensis*) as a primary food source for centuries [2]. United States Food and Drug Administration (FDA) also gave *Spirulina* the “Generally Recognized as Safe (GRAS)” status. Furthermore, the Expert Committee on Dietary Supplement Information of the United States Pharmacopeia Convention (USP) has classified *Spirulina* as ‘Class A’ for human consumption, deeming it safe when cultivated under controlled conditions. This conclusion comes after a rigorous analysis of clinical case reports, animal toxicological data, and adverse event reports [1,3,4,5]. The Food and Agriculture Organization of the United Nations (FAO) has named *Spirulina* a “highly digestible protein product,” and the US Space Agency has used it as a nutritional supplement for astronauts. Consequently, the World Health Organization (WHO) declared *Spirulina* as the best food of the future in 1996 due to its high protein and natural vitamin content [6,7].

Due to its high nutritional content and easy digestibility, *Spirulina* (*S. platensis*) serves as an excellent food source for humans and is also utilized as animal feed [6,7,8]. Its protein content consists of 60% to 70% of its dry weight, and it also contains polysaccharides, polyunsaturated fatty acids, carotenoids, B vitamins, and various minerals such as K, Mg, Zn, Na, Fe, Ca, Se, and Mn [4]. *Spirulina* also has an advanced nutritional profile with highly bioavailable components such as essential amino acids (64–74% of protein content), bili-proteins and essential polyunsaturated fatty acids including allophycocyanin, C-phycocyanin, *α*-chlorophyll, glycolipids, sulfo-lipids and *γ*-linoleic acid, vitamins B and E, and mineral substances [8,9]. These compounds are easily absorbed by the human body and help restore nutritional status to normal levels [10]. In addition to its nutritional properties, *Spirulina* has high therapeutic potential for the prevention and treatment of numerous health issues. Studies have proved antioxidant, immune-boosting, antidiuretic, hypocholesterolemic, anti-inflammatory, cardiovascular protective, and antiviral properties, along with its enhanced digestive capacity [11]. Most of the studies attribute its health-related benefits to antioxidant potential [12]. With the aim of benefiting from this potential, *Spirulina* has been incorporated into wide-scale food products from yogurt [13] to honey [14], from functional fermented whey-based sports beverages [15] to gluten-free biscuits [16].

Owing to its rich biochemical composition, *Spirulina* has attracted considerable interest not only as a direct food ingredient but also as a promising substrate for fermentation-based functional products.

Fermentation is a valuable bioprocess primarily used for food preservation by extending shelf life and improving the organoleptic and nutraceutical properties of food products [17]. According to Guinness World Records, evidence of early fermented beverages dating back to approximately 7000 B.C. was discovered in China [18]. During vinegar fermentation, microorganisms convert ethanol into acetic acid and simultaneously promote biochemical transformations that break down complex compounds into smaller molecules with improved digestibility and bioavailability [11]. Vinegar is one of the oldest fermented foods consumed worldwide and has traditionally been used as a preservative, flavoring agent, and functional food. Besides acetic acid, vinegar also contains various bioactive constituents such as organic acids, phenolic compounds, amino acids, and peptides, which are associated with its biological activities [19]. Previous studies have reported that vinegar exhibits antioxidant, antimicrobial, antihyperglycemic, and lipid-lowering properties [19]. These biological effects largely depend on the raw materials used and the biochemical transformations occurring during fermentation [20].

In this study, commercially available *Spirulina platensis*-based vinegar (SV) and apple vinegar (AV), along with *Spirulina platensis* powder (SP), were evaluated in terms of their organic acid and phenolic compound profiles, bioactive potential, and in vitro bioaccessibility. Additionally, the study aimed to elucidate the role of fermentation in shaping the bioactive composition and functional properties of SV.

## 2. Materials and Methods

### 2.1. Materials

Vinegar samples were obtained from an artisan-vinegar producer (Vinegral, Bursa, Türkiye). Apple fruits used in vinegar production were obtained from local organic farmers (Bursa, Türkiye). *Spirulina platensis* powder (SP) used in vinegar production was obtained from Hanna (Mugla, Türkiye).

### 2.2. Chemicals

All chemicals used in antioxidant capacity analysis, organic acid and phenolic compound standards were obtained from Merck (Darmstadt, Germany) and/or Sigma-Aldrich (St. Louis, MO, USA), respectively.

### 2.3. Methods

#### 2.3.1. Vinegar Production

Vinegar samples were obtained following the traditional fermentation method [20,21]. For apple vinegar (AV) production, 3.5 kg of organic apples were crushed and mixed with 10 L of sterilized drinking water. For SV production, SP was added at a concentration of 10 g/L to 10 L of sterilized drinking water. Traditional spontaneous vinegar fermentation was employed for vinegar production. Alcoholic fermentation was carried out under anaerobic conditions in airtight containers at room temperature (20–25 °C) and protected from direct sunlight. Following alcoholic fermentation, previously produced apple vinegar and vinegar mother (250 mL) were added to each container to initiate acetic acid fermentation. This stage was performed under aerobic conditions at room temperature (20–25 °C), with the containers covered by double-layer cheesecloth until the vinegar mother (pellicle) settled at the bottom. The vinegar samples were then transferred into hermetically sealed glass bottles and stored in the dark at 20 ± 2 °C until analysis. All analyses were performed in triplicate.

#### 2.3.2. Physico-Chemical Analysis

The total acidity of vinegar samples was determined according to AOAC [22] (Method No: 942.15) and expressed as acetic acid equivalent. pH values were determined according to AOAC [23] (Method No: 981.12; S220-K Seven Compact, Mettler Toledo, Milano, Italy).

#### 2.3.3. Organic Acid Determination

The organic acid contents of samples were identified according to the methodology described by Coelho et al. [24] with slight modifications as mentioned in Yildiz [20] with analytical method performance parameters. Sigma Aldrich Organic Acids Kit 47264 (St. Louis, MO, USA) which contains D-(+)-malic, citric, propionic, succinic, oxalic, L-(+)-lactic, benzoic, fumaric, D-(−)-tartaric, adipic, maleic, shikimic, acetic, formic, phytic, and (−)-quinic acids, as well as butyric, dL-isocitric, malonic, isobutyric, and L-ascorbic acid was utilized as analytical standards. HPLC (1260 Infinity LC model, Agilent Technologies, Santa Clara, CA, USA) was equipped with DAD (Diode array detector) (1260, G1315C model, Agilent, Santa Clara, CA, USA), and an ion exchange column was used (300 × 7.7 mm, 8 µm; Hi-Plex H, AGPL1170-6830, Agilent, Santa Clara, CA, USA) for separation of organic acids. The mobile phase was 0.02 N H_2_SO_4_ with a 0.6 mL/min flow rate (50 °C, 36.5 bar detector pressure), and the injection volume was 10 μL for an isocratic operational system. All measurements were performed in triplicate.

#### 2.3.4. Phenolic Compound Determination

The phenolic compound contents of samples were identified according to the methodology described by Selli [25], as detailed in Yildiz [20] in terms of quercetin, protocatechuic acid, rutin, ferulic acid, catechin, neohesperidin, gallic acid, trans-cinnamic acid, vanillic acid, hesperidin, *p*-coumaric acid, alizarin, gentisic acid, resveratrol, hydroxybenzoic acid, flavone, ascorbic acid, naringin, *o*-coumaric acid, and coumarin. HPLC (1260 Infinity LC model, Agilent Technologies, Santa Clara, USA) was equipped with a diode array detector (DAD) (1260, G1315C model, Agilent, USA), and a C_18_ column (250 × 4.6 mm, 5 μm; ACE Generix^®^, Advanced Chromatography Technologies, Aberdeen, UK) was used for the separation. Mobile phase A was phosphoric acid solution (0.1%, v/v), and phase B was acetonitrile. The flow rate was 0.8 mL/min (30 °C, 36.5 bar detector pressure), and the injection volume was 10 μL. All measurements were performed in triplicate.

#### 2.3.5. Antioxidant Capacity and Total Phenolic Content

FRAP (Ferric Reducing Antioxidant Power Assay), DPPH (2,2-diphenyl-1-picrylhydrazyl) and ABTS (2,2-azinobis-[3-ethylbenzothiazoline-6-sulphoni-cacid]), CUPRAC (Cupric Reducing Antioxidant Capacity) antioxidant capacity (AC) assays and total phenolic content (TPC, Folin–Ciocalteu’s method) analysis were utilized for the determination of bioactive potential. Extractable phenolic fraction (EPF) and hydrolyzable phenolic fraction (HPF) were obtained according to Vitali et al. [26], and bioaccessible phenolic fraction (BPF) was obtained according to Bouayed et al. [27] by in vitro enzymatic digestion extraction. All obtained extracts were stored at −20 °C until the analyses.

FRAP AC assay was performed according to Benzie and Strain [28]. 0.02–0.08 µmol TB calibration curve (y = 52.834x + 0.1005, R^2^ = 0.9997) was utilized in calculations (Blank: methanol). DPPH AC assay was performed according to Brand-Williams et al. [29]. 0.02–0.08 µmol TB calibration curve (y = 3246.2x + 0.7181, R^2^ = 0.9941) was utilized in calculations (Blank: methanol). ABTS AC assay was performed according to Apak et al. [30]. 0.02–0.08 µmol Trolox^®^-based (TB) calibration curve (y = 3804.9x − 3.8599, R^2^ = 0.9969) was utilized in calculations (Blank: ethanol). CUPRAC AC assay was performed according to Apak et al. [30]. 0.02–0.08 µmol TB calibration curve (y = 12.015x − 0.0013, R^2^ = 0.9985) was utilized in calculations (Blank: methanol). All AC assay results (Triplicated) were expressed as µmol Trolox^®^ equivalents (TE) per g-mL sample.

The TPC was determined by Naczk and Shahidi [31]. 10–500 mg/L gallic acid (GA)-based calibration curve (y = 0.006x − 0.0055, R^2^ = 0.9996) utilized in calculations (Blank: distilled water). TPC results were expressed as mg gallic acid equivalent (GAE)of g-mL per g-mL of sample.

The bioaccessibility percentage (B%) of AC and total phenolic content (TPC) was calculated in order to estimate the proportion of phenolic compounds available after simulated digestion. The calculation was based on the results obtained from EPF, HPF, and BPF using the following Equation (1) [32].

(1) $$B\%=\frac{BPF}{EPF+HPF}\times 100$$

#### 2.3.6. Statistical Analysis

Statistical analyses were performed using SPSS software (version 21; SPSS Inc., Chicago, IL, USA). Data were expressed as mean ± standard deviation, and measurements were performed in triplicate. Differences between vinegar samples were evaluated using a t-test at a significance level of *p* < 0.05. Due to differences in matrix and units, SP was not included in the statistical comparison and was presented for descriptive purposes only.

Principal component analysis (PCA) was performed using SPSS Statistics (version 21; SPSS Inc., Chicago, IL, USA) based on the correlation matrix. Prior to analysis, variables were standardized (z-scores) to account for differences in scale and units. Components with eigenvalues greater than 1 were retained, and varimax rotation was applied to improve interpretability. The analysis included all measured variables (organic acids, phenolic compounds, and AC parameters such as TPC, DPPH, ABTS, and FRAP).

## 3. Results and Discussion

### 3.1. pH and Total Acidity

Acidity development is the most important parameter of the fermentation for the sensory and quality criteria of vinegar. *Spirulina platensis*-based vinegar (SV) had 5.75 g/100 mL total acidity, while apple vinegar (AV) had 5.05 g/100 mL as acetic acid equivalent. The higher acidity observed in SV may be associated with the biochemical composition of *Spirulina*, which provides readily available nutrients that can enhance microbial metabolism during fermentation and promote more efficient ethanol oxidation by acetic acid bacteria (AAB). Consistent with the acidity values, pH was determined as 2.92 and 2.97, respectively. Vinegar must have an acidity content of at least 4.00 g/100 mL in terms of acetic acid [33]. The carbohydrate content and quality of the raw materials contribute to higher acidity development in vinegars. Organic and ripe fruits have been determined to accelerate fermentation and increase the overall acid content in vinegar production [20]. Kong et al. [34] noted that the type of AAB and fermentation temperature significantly influence acid production capacity and the formation of volatile aroma compounds. Acid-tolerant species, such as Komagataeibacter and *Acetobacter pasteurianus*, in particular, produced higher acidity values and an improved aroma profile. While the titratable acidity in vinegar samples obtained from a single AAB culture was 5.53 ± 0.13%, this value increased to 6.51 ± 0.44% in multiple cultures. This suggests that microbial diversity increases fermentation efficiency. They also reported that synergistic metabolic interactions directly related to the beta-diversity (*β*-diversity) of the microbial community support acid production and that increased AAB activity in systems with adequate oxygen transfer leads to significant improvements in the sensory and functional quality of the product. These findings support the results of the present study, where differences in substrate composition may have contributed to the observed variation in acidity between SV and AV.

### 3.2. Organic Acid Content

**Figure 1:**
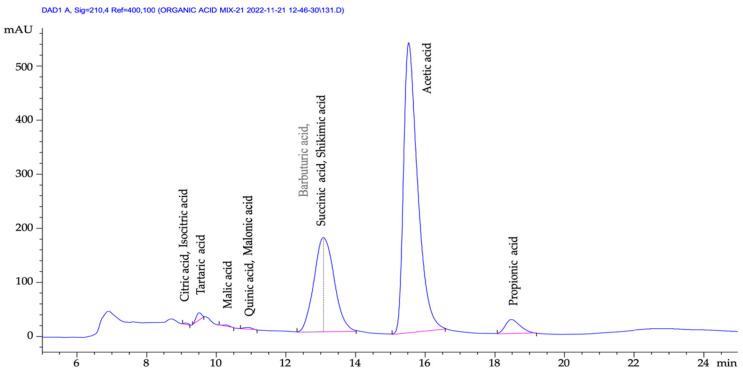
Organic acid chromatogram of *Spirulina platensis*-based vinegar (Signal 210,4 nm Ref; 400,100 nm).

Organic acid profiles of SP and vinegar samples (SV and AV) are presented in Table 1, with the corresponding chromatograms shown in Figure 1. Vinegar fermentation involves two sequential biological processes: alcoholic fermentation followed by acetic acid fermentation. The tricarboxylic acid (TCA) cycle represents a central metabolic pathway involved in the utilization of carbohydrates, lipids, and amino acids during vinegar fermentation. Organic acids naturally present in vinegar can participate in these energy-generating metabolic reactions. In this process, AAB metabolizes available carbon sources to synthesize various organic acids, while simultaneous protein degradation contributes to the release of amino acids. Furthermore, certain amino acids and fatty acids may undergo oxidative catabolism through TCA-associated pathways, resulting in the generation of low-molecular-weight compounds [35,36]. The chemical composition of the raw materials influences fermentation performance and acetic acid and organic acid production. Considering acetic acid as the primary indicator of fermentative acidity, SV exhibited a higher content (58.05 g/L) than AV (48.64 g/L). In fruit vinegars such as apple cider vinegar, higher carbohydrate content is generally associated with increased vinegar yield [20,36]. Gullo and Giudici [37] reported that the growth and metabolic activity of acetic acid bacteria (AAB) are influenced by nitrogen sources. Known for its high available amino acid content [8,9], SP may increase the microbial activity and improve the acetic acid and organic acid profile of SV.

Oxalic-dihydrate (6.27 ± 0.02 mg/L), adipic acid (934.21 ± 1.21 mg/L), L-(+)-lactic acid (39.85 ± 0.05 mg/L), and butyric acid (1.45 ± 0.02 mg/L) were detected only in AV and were not found in either SP or SV. Therefore, these compounds can be considered characteristic organic acids of the AV. Also, the phytic acid was determined only in SP (494.43 ± 5.57 mg/L, Table 1), suggesting that fermentation provided the degradation of this compound. During fermentation, certain bacteria and yeasts are known to produce phytase enzymes that hydrolyze phytic acid into lower phosphorylated inositol derivatives [38]. Also, phytase activity increases under acidic conditions, such as during vinegar fermentation [39]. Vinegar fermentation is an important biotechnological process that reduces antinutritional components like phytic acid and seems to enhance the functional quality of foods, even in substrates like *Spirulina* powder.

SP contributed to vinegar fermentation in terms of its organic acid profile; in particular, the presence of DL-isocitric, shikimic, citric, D-(+)-malic and succinic acids enriched the organic acid composition of the SV sample compared to AV. Among these, shikimic and succinic acids were the most prominent, with significantly higher levels observed in SV (*p* < 0.05, Table 1). Previously, shikimic acid content has been determined in red fruit vinegars between 2.87 ± 0.08 and 17.67 ± 0.02 mg/L [20], whereas SV exhibited a markedly higher concentration of 147.13 ± 1.37 mg/L, indicating a substantial enrichment. Rabelo et al. [40] reported shikimic acid as having a neuroprotective effect against oxidative stress induced by hydrogen peroxide, and Li et al. [41] stated that 50 mg/kg shikimic acid can support the recovery of intestinal flora imbalance and protect intestinal health. In light of the findings by Rabelo et al. [40] and Li et al. [41], SV demonstrated a high shikimic acid content of 147.13 ± 1.37 mg/L, suggesting significant potential. Tartaric acid, as a commonly determined organic acid in vinegar samples, has remarkable content (146.64 ± 1.12 mg/L), (*p* < 0.05) in SV, compared to AV (28.31 ± 0.45 mg/L). It has been determined to be effective on blood pressure and has vasodilatory properties [42]. SV suggested reflecting more antihypertensive potential effect, comparing it to AV.

Quinic acid, detected as 33.38 ± 0.39 mg/kg in SP and 125.74 ± 0.46 mg/L in SV (not detected in AV), can be considered SP’s contribution to SV as an enriching component. Additionally, lactic acid was detected only in AV (39.85 ± 0.05 mg/L) and was absent in SV. Xia et al. [43] reported that lactic acid bacteria (LAB) produce lactic acid during the early stages of fermentation; however, as the environment becomes more acidic and oxygenated, LAB activity declines due to the dominance of acetic acid bacteria (AAB). This microbial shift likely contributes to the absence of lactic acid in SV. Furthermore, AAB may utilize lactic acid as a substrate during acetification, influencing the overall organic acid profile. Maleic, formic, and isobutyric acids were not detected in any of the samples.

### 3.3. Phenolic Compound Content

**Figure 2:**
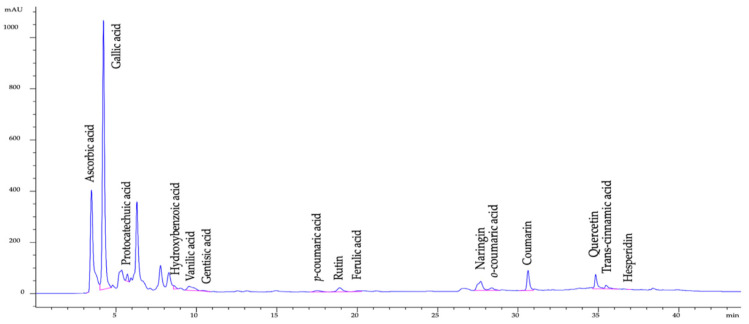
Phenolic compound chromatogram of the *Spirulina plantensis*-based vinegar (Signal 300,200 nm Ref; 500,100 nm).

Individual phenolic compound profiles of samples are given in Table 2. (The corresponding chromatogram is given in Figure 2). Phenolic compounds are presented in Table 2 according to their structural complexity, facilitating the evaluation of the fermentation process [44]. Phenolic compounds proved to exhibit characteristic effects on the bioactive potential, as well as the volatile profile, aromatic properties and quality characteristics of vinegars [36,45,46].

Protocatechuic acid, *p*-coumaric acid, rutin, ferulic acid, trans-cinnamic acid, and hesperidin were found at significantly higher levels in AV than in SV (*p* < 0.05). Quercetin was detected in all three samples, with a higher concentration in SV (2.29 ± 0.01 mg/L) compared to AV (1.56 ± 0.01 mg/L). o-coumaric acid was not detected in SP but was present in AV (0.54 ± 0.00 mg/L) and SV (0.76 ± 0.27 mg/L), with higher levels observed in SV. Resveratrol was not detected in any of the samples. Gallic acid was significantly more abundant in SV (86.99 ± 0.14 mg/L) than in AV (9.59 ± 0.06 mg/L), while its level in SP was low (0.99 ± 0.01 mg/kg). In comparison, red fruit vinegars have been reported to contain approximately twice as much gallic acid as AV [20], whereas SV exhibited nearly tenfold higher levels (Table 2). These findings suggest that vinegar fermentation may have contributed to an increase in gallic acid content. Gallic acid is a low-molecular-weight phenolic acid with relatively higher bioavailability compared to more complex phenolic structures [44]. Previous studies have linked changes in phenolic profiles during vinegar fermentation—particularly increases in gallic acid—to the activity of tannin-hydrolyzing enzymes (tannase) [47]. Similarly, Tang et al. [48] associated tannase activity with the release of functional phenolics during fermentation processes, including wine and vinegar. Conversely, the decrease in certain compounds may be attributed to their utilization as substrates in microbial metabolism or to oxidative degradation processes [47].

During fermentation, phenolic compounds may undergo enzymatic biotransformation through microbial activities such as hydrolysis, decarboxylation, and oxidation. These changes appear to be compound-specific rather than uniform. In the present study, certain phenolics, including gallic acid, quercetin, coumarin, and naringin, showed increases, whereas others, such as protocatechuic, vanillic, and ferulic acids, as well as rutin and hesperidin, decreased. This pattern suggests that microbial metabolism may selectively transform phenolic compounds depending on their chemical structure and susceptibility to enzymatic reactions [49]. Overall, these findings suggest that vinegar fermentation may act not only as an acetic acid production process but also as a transformation step that potentially enhances the functional properties of the product through phenolic biotransformation.

The higher quercetin content observed in SV (Table 2) may suggest that phenolic biotransformation occurs during fermentation, potentially contributing to the functional properties of the product [47]. It has been reported that Fe-dependent quercetin 2,3-dioxygenase (quercetinase) catalyzes the oxidative cleavage of the flavonol C-ring, leading to ring-opening products; through these pathways, protocatechuic acid may be further converted into 4-hydroxybenzoic derivatives [49]. Accordingly, the variation in quercetin and protocatechuic acid levels observed between SV and AV may reflect differences in metabolic pathways during fermentation. Additionally, microbial or enzymatic modifications, such as O-methylation, may alter phenolic ring structures, leading to the formation of methylated phenolic acids (e.g., conversion of vanillic acid to ferulic acid [50]. Jin et al. [47] pictured well three effective mechanisms: Coenzyme A (CoA)-independent oxidative pathway, CoA-dependent non-oxidative pathway and CoA-dependent β-oxidative pathway. Also, higher citric acid and isocitric acid content in SV is suggested to be involved in these transformations. Naringin and hesperidin were also known to take part in quercetin formation [47]. Compared with conventional substrates used for vinegar production, SP is characterized by relatively high levels of C18:2 (linoleic acid, LA; 144.81 ± 18.76 mg/100 g) and C18:3 (γ-linolenic acid, GLA; 1866.27 ± 37.37 mg/100 g) [14]. Zhang et al. [51] produced a GLA-enriched rose vinegar using mixed cultures (*Mucor circinelloides* and LAB) and associated its bioactive potential (TEAC-DPPH) with phenolic compounds such as gallic acid and vanillic acid. Similarly, the elevated gallic acid content observed in SV may be partially associated with microbial activity, including that of LAB.

When all the obtained values are evaluated together, the gallic acid content is the most noteworthy output for SV (Table 2), in terms of phenolic compounds. These observations suggest that AV and SV may follow distinct phenolic transformation pathways.

### 3.4. Bioactive Potential

Based on the data presented in Table 3, SV exhibited significantly higher bioactive potential than both SP and AV across all AC assays. This trend was consistently observed across assays based on different reaction mechanisms, suggesting the robustness of the results.

ABTS and DPPH assays, which involve both hydrogen atom transfer (HAT) and single electron transfer (SET) mechanisms, evaluate the radical scavenging capacity of antioxidants [28,29,47]. FRAP and CUPRAC assays assess reducing power and are predominantly based on electron transfer (SET) mechanisms [30,52,53]. While FRAP reflects Fe^3+^ → Fe^2+^ reduction and is more selective for strong reductants, CUPRAC (Cu^2+^ → Cu^+^) is responsive to a broader range of phenolic compounds [28,53]. The higher values observed for SV may be associated with the presence of low-molecular-weight and more reactive phenolic compounds, organic acids formed during vinegar fermentation and their synergistic and antagonistic behavior with each other [54,55,56].

In vinegar samples, the elevated TEAC_FRAP_ and TEAC_CUPRAC_ values compared to TEAC_ABTS_ and TEAC_DPPH_ values in SV were suggested to be linked with the increased diversity and concentration of reducing compounds. Higher gallic acid content of the SV (compared to AV) was suggested to be the most associated content, with higher TEAC_FRAP_ and TEAC_CUPRAC_ results together with synergistic interactions of phenolic compounds and fermentation-produced organic acids [56]. Phenolic acids, including gallic acid, may also contribute to the overall functional properties of the product. These compounds have been reported to interact with microbial metabolism by modulating oxidative stress and stabilizing fermentation processes under acidic conditions [47]. For EPF, HPF and BPF, the bound phenolic structures present in SP may be broken down into simpler free phenolic acids, especially gallic acid, through microbial enzyme activity and the influence of the acidic environment during the fermentation. This transformation may increase the proportion of simpler phenolic compounds, potentially enhancing their bioavailability. Furthermore, the increase in simple phenolics such as gallic acid may contribute to the AC of the sample. Skroza et al. [54] investigated the synergistic and antagonistic effects of phenolic compounds in terms of FRAP and CUPRAC AC assays and determined that gallic acid showed the highest, while vanillic acid showed lower TEAC_FRAP_ values (higher gallic acid content observed in SV, Table 2); also, ferulic acid + *p*-coumaric acid exhibited the lowest results (higher ferulic and *p*-coumaric acid contents observed in AV, Table 2). Furthermore, Apak et al. [55] determined that gallic acids showed higher TEAC_CUPRAC_ results, stating that the degree of conjugation of the molecule, as well as the number and group of hydroxyl groups, were effective in terms of electron transfer. In this context, not only the quantity of individual bioactive components but also their synergistic and antagonistic behavior towards each other is important from a bioactivity perspective. Furthermore, the chosen AC assay for determining bioactive potential is also crucial in terms of the results obtained and the reflection of the potential of the evaluated samples. In addition, TPC results were correlated with AC assay values. Liu et al. [57] also determined a similar relation with TEAC and FRAP AC assay values with TPC values in fruit vinegars in the same context.

Overall, the fermentation process was suggested to enhance AC (as EPF, HPF, and BPF), as evaluated by both radical scavenging and reducing power assays, potentially through the conversion of bound phenolic compounds in the SP matrix into simpler and more reactive forms. This suggests that fermentation may play a key role in improving the concentration and bioaccessibility of bioactive compounds.

### 3.5. Principal Component Analysis Evaluation

**Figure 3:**
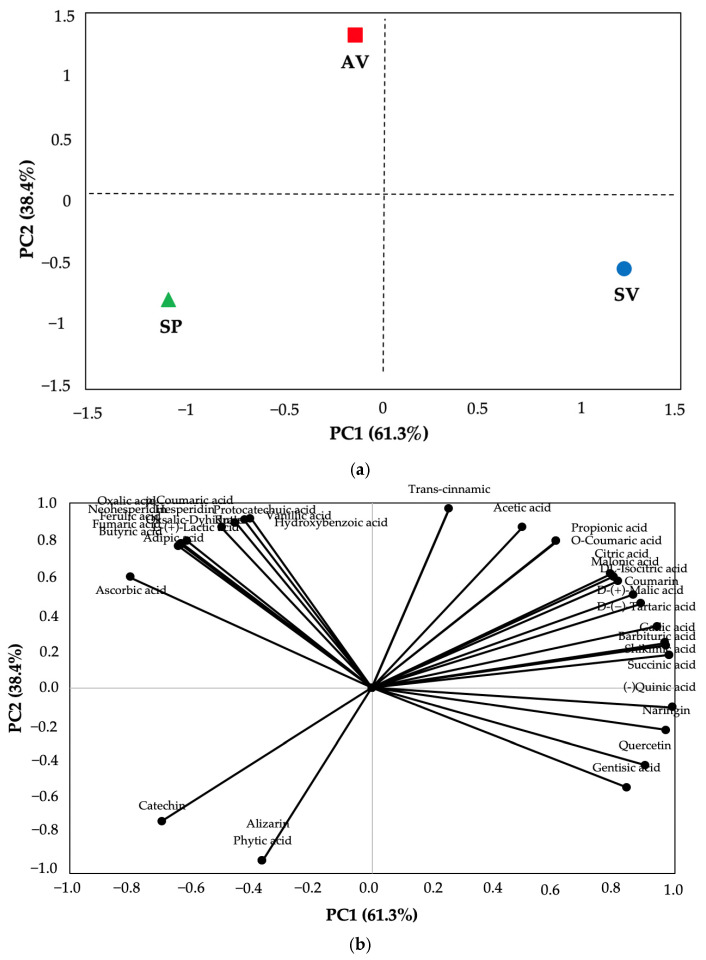
(**a**). PCA score plot based on organic acids and phenolic compounds of *Spirulina platensis* powder (SP), *Spirulina platensis*-based vinegar (SV), and apple vinegar (AV). (**b**). PCA loading plot based on organic acids and phenolic compounds.

PCA was performed based on organic acids and phenolic compounds to evaluate compositional differences among SP, AV, and SV (Figure 3a,b).

The PCA results provided an integrated interpretation for the compositional changes in the vinegars during fermentation. The principal component PC1 (61.3%) was mainly affected by the variables with high loading values, including total organic acids and AC parameters, such as TPC (0.995), DPPH (0.996), ABTS (0.996) and FRAP (0.969), and major organic acids, such as malic (0.998), tartaric (0.995), citric (0.978) and succinic acid (0.967) in the separation of the samples. These results suggest that this axis can be interpreted as reflecting overall biochemical enrichment of the matrix. This observation is consistent with the higher levels of acetic, succinic, shikimic, and other organic acids in SV, as well as the enhanced AC observed across assays. The clustering of these variables points to vinegar fermentation’s contribution to the provision of bioactive compounds and the combined bioactive potential as reported in complex fermented matrices [52]. On the contrary, PC 2 (38.4%) was correlated to specific organic acids and individual phenolic compounds with high loading contributions, such as oxalic (0.971), lactic (0.971), and fumaric acids (0.971), as well as gallic acid (0.989) and coumarin (0.991), and discriminated samples based on compositional differences, rather than quantitative ones. This pattern is in accordance with the changes in phenolic compounds, where complex structures could be enzymatically converted to simpler phenolics such as gallic acid, and other phenolics might be reduced by microbial metabolism. The distinct positioning of SV along PC2 reflects this biotransformation-related shift in phenolic profile, rather than a uniform change across all compounds. Microbial metabolism can lead to similar compositional differentiation in vinegar and other fermented matrices, with interactions between microorganisms influencing both the profile of organic acids and the changes in phenolic compounds [35,43,48].

The PCA results indicated that phenolic compounds played an important role in sample discrimination, particularly along PC2, which was associated with higher levels of compounds such as gallic acid and quercetin (loading values: 0.989 and 0.673, respectively). These findings further suggest that PC1 reflects the cumulative contribution of organic acids and bioactive potential-related compounds, whereas the qualitative distribution of individual phenolics and organic acids is critical in defining product-specific characteristics. Therefore, the differentiation between SV and AV, rather than the individual placement of SP, may be attributed to the higher bioactive content and compositional modifications generated during fermentation. This observation highlights the dual role of fermentation as both an enrichment and transformation process.

Since this study utilized only a single fermentation batch per sample type, additional research involving multiple independent fermentation batches is necessary to validate the reproducibility and robustness of the findings. Obtained results should be evaluated in this consideration.

## 4. Conclusions

This study evaluated the use of Spirulina powder (SP) in traditional vinegar fermentation. *Spirulina platensis*-based vinegar (SV) exhibited a higher bioactive potential due to its organic acid composition and phenolic profile, compared to apple vinegar (AV). The higher contents of acetic, succinic and shikimic acids, as well as the increase in gallic acid, indicate that fermentation based on SP can lead to compositional enrichment and functional improvement. Rather than a simple increase or decrease in phenolic compounds, the findings indicate that fermentation may induce structural transformations, converting more complex phenolics into simpler and potentially more bioaccessible forms. The differences observed between SV and AV also suggest the effect of the substrate type on the metabolic pathways and the resulting bioactive composition. The results of the PCA indicate a significant impact of organic acids on the overall bioactive potential, while phenolic compounds seem to be the key drivers for the compositional discrimination of the samples. Ultimately, *Spirulina platensis* has the potential to be a promising substrate for the production of vinegar, which could contribute to the development of functionally enriched products with enhanced bioactive properties. It should be noted that the findings are based on an in vitro approach, which may limit the direct interpretation of functional effects. Therefore, further studies incorporating cell-based assays and in vivo models are required to confirm the biological relevance and functional properties of SV.

## Figures and Tables

**Table 1:** Organic acid profiles of samples.

Organic Acid	*Spirulina platensis* Powder (SP, mg/kg)	Apple Vinegar (AV, mg/L)	*Spirulina platensis* Vinegar (SV, mg/L)	LOD (µg/kg)	LOQ (µg/kg)
Malonic acid	Nd.	31.62 ± 0.81 ^b^	64.80 ± 0.27 ^a^	102.55	341.85
Barbituric acid	Nd.	3.48 ± 0.12 ^b^	41.02 ± 0.75 ^a^	1.35	4.50
Phytic acid	494.43 ± 5.57	Nd.	Nd.	162.11	540.36
Oxalic-Dyhidrate	Nd.	6.27 ± 0.02	Nd.	7.29	24.30
DL-Isocitric acid	3.33 ± 0.70	4.54 ± 0.03 ^b^	6.05 ± 0.04 ^a^	14.75	49.16
(-)Quinic acid	33.38 ± 0.39	Nd.	125.74 ± 0.46	83.24	277.47
Shikimic acid	0.19 ± 0.00	4.34 ± 0.06 ^b^	147.13 ± 1.37 ^a^	1.10	3.66
Adipic acid	Nd.	934.21 ± 1.21	Nd.	131.47	438.24
Oxalic acid	Nd.	3.99 ± 0.03	Nd.	6.39	21.29
Citric acid	15.51 ± 0.01	23.80 ± 0.14 ^b^	33.05 ± 0.29 ^a^	3.96	13.20
D-(−)-Tartaric acid	Nd.	28.31 ± 0.45 ^b^	146.64 ± 1.12 ^a^	14.37	47.90
D-(+)-Malic acid	21.32 ± 0.16	32.05 ± 0.59 ^b^	54.78 ± 1.49 ^a^	16.77	55.90
Succinic acid	17.48 ± 0.04	462.36 ± 0.64 ^b^	15,859.43 ± 147.24 ^a^	70.11	233.70
L-(+)-Lactic acid	Nd.	39.85 ± 0.05	Nd.	43.86	146.20
Acetic acid	Nd.	48,694.17 ± 191.02 ^b^	58,056.00 ± 203.34 ^a^	22.05	73.50
Fumaric acid	Nd.	0.42 ± 0.01	Nd.	18.48	61.60
Propionic acid	Nd.	148.74 ± 0.08 ^b^	209.86 ± 1.06 ^a^	26.16	87.02
Butyric acid	Nd.	1.45 ± 0.02	Nd.	32.86	109.54

Values are given as mean ± SD. Nd.: Not detected. Different letters within the same row indicate significant differences between vinegar samples (*p* < 0.05). SP was not included in statistical comparisons and is presented for descriptive purposes only.

**Table 2:** Phenolic compound profiles of samples.

Phenolic Compound	*Spirulina platensis* Powder (SP, mg/kg)	Apple Vinegar (AV, mg/L)	*Spirulina platensis* Vinegar (SV, mg/L)
Hydroxybenzoic acid	Nd.	2.91 ± 0.01 ^a^	0.73 ± 0.00 ^b^
Gallic acid	0.99 ± 0.01	9.59 ± 0.06 ^b^	86.99 ± 0.14 ^a^
Protocatechuic acid	0.12 ± 0.01	5.30 ± 0.01 ^a^	1.00 ± 0.02 ^b^
Gentisic acid	0.17 ± 0.01	Nd.	0.25 ± 0.01
Vanillic acid	Nd.	5.88 ± 0.01 ^a^	1.60 ± 0.00 ^b^
*p*-coumaric acid	0.02 ± 0.00	4.80 ± 0.01 ^a^	0.14 ± 0.00 ^b^
*o*-coumaric acid	Nd.	0.54 ± 0.00 ^b^	0.76 ± 0.27 ^a^
Trans-cinnamic	0.06 ± 0.00	1.28 ± 0.01 ^a^	1.13 ± 0.01 ^b^
Ferulic acid	0.40 ± 0.01	23.67 ± 0.23 ^a^	0.27 ± 0.00 ^b^
Ascorbic acid	0.03 ± 0.01	0.13 ± 0.00	Nd.
Coumarin	0.09 ± 0.00	1.11 ± 0.02 ^b^	2.84 ± 0.01 ^a^
Alizarin	17.02 ± 0.04	Nd.	Nd.
Catechin	0.70 ± 0.01	0.27 ± 0.01	Nd.
Quercetin	1.97 ± 0.07	1.56 ± 0.01 ^b^	2.29 ± 0.01 ^a^
Rutin	0.77 ± 0.01	5.30 ± 0.23 ^a^	1.76 ± 0.01 ^b^
Naringin	2.09 ± 0.01	Nd.	5.49 ± 0.03
Neohesperidin	Nd.	0.63 ± 0.01	Nd.
Hesperidin	0.06 ± 0.00	1.51 ± 0.25 ^a^	0.03 ± 0.00 ^b^

Values are given as mean ± SD. Nd.: Not detected. Different letters within the same row indicate significant differences between vinegar samples (*p* < 0.05). SP was not included in statistical comparisons and is presented for descriptive purposes only.

**Table 3:** Bioactive potential of samples.

AC Assay	*Phenolic* *Fraction*	*Spirulina platensis* Powder	Apple Vinegar	*Spirulina platensis* Vinegar
ABTS µmol TE/g-mL	EPF	1.42 ± 0.01	5.48 ± 0.03 ^b^	21.76 ± 0.24 ^a^
	HPF	3.43 ± 0.01	3.74 ± 0.04 ^b^	9.25 ± 0.09 ^a^
	BPF	4.38 ± 0.01	8.68 ± 0.04 ^b^	25.68 ± 0.06 ^a^
	B%	*90.24 ± 0.23 ^B^*	*94.14 ± 0.72 ^A^*	*82.80 ± 5.01 ^C^*
CUPRAC µmol TE/g-mL	EPF	1.82 ± 0.01	3.74 ± 0.25 ^b^	10.99 ± 0.23 ^a^
	HPF	6.00 ± 0.01	1.43 ± 0.0 ^b^	5.52 ± 0.06 ^a^
	BPF	7.14 ± 0.02	3.55 ± 0.23 ^b^	9.90 ± 0.46 ^a^
	B%	*91.18 ± 0.22 ^A^*	*65.36 ± 4.47 ^B^*	*68.69 ± 2.24 ^B^*
DPPH μmol TE/g-mL	EPF	1.66 ± 0.03	10.62 ± 0.16 ^b^	44.60 ± 0.57 ^a^
	HPF	4.38 ± 0.01	5.58 ± 0.00 ^b^	16.44 ± 0.23 ^a^
	BPF	3.68 ± 0.01	12.94 ± 0.17 ^b^	35.00 ± 0.09 ^a^
	B%	*61.05 ± 0.37 ^B^*	*79.86 ± 0.40 ^A^*	*57.36 ± 0.77 ^C^*
FRAP µmol TE/g-mL	EPF	3.20 ± 0.01	3.76 ± 0.10 ^b^	18.11 ± 0.03 ^a^
	HPF	6.64 ± 0.00	5.63 ± 0.03 ^b^	31.47 ± 0.03 ^a^
	BPF	5.38 ± 0.01	6.81 ± 0.08 ^b^	22.35 ± 0.05 ^a^
	B%	*54.60 ± 0.38 ^B^*	*72.60 ± 0.87 ^A^*	*45.07 ± 0.06 ^C^*
TPC mg GAE/100 g-mL	EPF	11.65 ± 0.01	24.37 ± 0.39 ^b^	77.97 ± 0.39 ^a^
	HPF	20.65 ± 0.02	16.25 ± 0.08 ^b^	52.23 ± 1.11 ^a^
	BPF	13.58 ± 0.13	20.51 ± 0.72 ^b^	67.37 ± 1.98 ^a^
	B%	*42.03 ± 0.38 ^B^*	*50.52 ± 2.32 ^A^*	*51.75 ± 1.27 ^A^*

AC: Antioxidant capacity; TPC: Total phenolic content; EPF: Extractable phenolic fraction; HPF: Hydrolyzable phenolic fraction; BPF: bioaccessible phenolic fraction. Values are given as mean ± SD. Different lowercase letters (a,b) within the same row indicate significant differences between vinegar samples (*p* < 0.05). Different capital letters (A–C) represent statistical differences in B% values between samples (*p* < 0.05). % bioaccessibility values are given in italic that calculated from EPF, HPF, and BPF values according to Equation (1).

## Data Availability

The original contributions presented in this study are included in the article. Further inquiries can be directed to the corresponding author.
